# Beneficial effect of retigabine on memory in rats receiving ethanol

**DOI:** 10.1007/s43440-020-00205-z

**Published:** 2020-12-31

**Authors:** Ewa Zwierzyńska, Agata Krupa-Burtnik, Bogusława Pietrzak

**Affiliations:** Department of Pharmacodynamics, Medical University of Lodz, Muszyńskiego 1, 90-151 Łódź, Poland

**Keywords:** Retigabine, Ethanol, Memory, Rats

## Abstract

**Background:**

Retigabine belongs to the novel generation of antiepileptic drugs but its complex mechanism of action causes that the drug might be effective in other diseases, for instance, alcohol dependence. It is known that ethanol abuse impaired the function of brain structures associated with memory and learning such as the hippocampus. In our previous study, retigabine reduced hippocampal changes induced by ethanol in the EEG rhythms in rabbits. This study is focused on the impact of retigabine on memory processes in male rats receiving alcohol.

**Methods:**

Memory was evaluated in various experimental models: Morris water maze, Contextual, and Cued Fear Conditioning tests. Retigabine was administered for 3 weeks directly to the stomach via oral gavage at a dose of 10 mg/kg. Rats received also 20% ethanol (5 g/kg/day in two doses) via oral gavage for 3 weeks and had free access to 5% ethanol in the afternoon and at night. Morris water maze was performed after 1 and 3 weeks of ethanol administration and after 1 week from the discontinuation of ethanol administration. Contextual and Cued Fear Conditioning tests were carried out after 24 h and 72 h of alcohol discontinuation.

**Results:**

The drug significantly decreased ethanol-induced memory disturbances during alcohol administration as well as slightly improved learning processes after the discontinuation of ethanol administration.

**Conclusions:**

This beneficial effect of retigabine-ethanol interaction on memory may be a relevant element of the drug’s impact on the development of addiction.

## Introduction

The new generation of antiepileptic drugs is a complex group characterized by multidirectional mechanisms of actions. For this reason, efforts are made to assess the possibility of their use in the therapy of neurological disorders other than epilepsy. Retigabine is one of the most recent drugs belonging to antiepileptics and it has a unique mechanism of action associated mainly with the activation of voltage-gated potassium channels Kv7 [1]. The drug also increases GABAergic transmission, primarily through GABA_A_ receptors [2], inhibits glutamatergic transmission [3], and if administered at high doses, it blocks voltage-gated sodium and calcium channels [4]. Results of recent research indicate that retigabine may also inhibit striatal cholinergic interneuron activity [5].

It has been demonstrated that retigabine might effectively alleviate neuropathic pain [6] and be useful in dystonia and dyskinesia [7, 8], Parkinson’s disease [5], amyotrophic lateral sclerosis [9], bipolar disorder [10] or alcohol dependence [11]. Alcohol abuse is associated with an adverse effect on brain structures such as the hippocampus or cortex which correlates with memory and learning disorders [12]. We observed in our previous pharmaco-EEG study that retigabine decreased hippocampal ethanol-induced changes in the EEG rhythms in rabbits [13]. These results prompted us to continue research and this study is focused on assessing the interaction of ethanol and retigabine and their impact on memory processes related to the hippocampus. The aim of the study was to evaluate the influence of retigabine on memory processes not only during prolonged exposure to ethanol but also after discontinuation of its use. The impact of the drug on spatial memory was studied in rats using the Morris water maze task (MWM) while the effect on the memory associated with anxiety was tested after the discontinuation of ethanol administration in the Contextual Fear Conditioning (CFC) and Cued Fear Conditioning (CuFC) tests.

## Materials and methods

### Animals

In the study, 71 male Wistar rats (Medical University of Lodz, Poland) weighing 250–280 g were used. The animals were housed under normal laboratory conditions (20–22 °C, 12 h light/12 h of dark cycle) and had free access to commercial chow. Experiments were performed between 8:00 a.m. and 4:00 p.m. in the light phase.

In the MWM, rats were divided into three groups: control, ethanol and experimental (retigabine + ethanol). The doses of ethanol were based on the scheme of alcohol administration by Majchowicz [14] with Szmigielski’s amendment [15]. The forced ethanol intake model was used to eliminate the risk that the potential observed changes will be caused by a retigabine-induced reduction in alcohol consumption. Ethanol was administered for 3 weeks. Experimental and ethanol groups (respectively, *n* = 7, *n* = 8) received 20% ethanol *po *via an oral gavage twice a day in two doses: 1.5 g/kg (0.75 ml/100 g; morning) and 3.5 g/kg (1.75 ml/100 g; afternoon). Additionally, these animals had free access only to 5% ethanol between 4.00 p.m. and 8.00 a.m. In turn, water was available for free drinking only from 8 a.m. to 4 p.m. The third group of animals (control, *n* = 7) received water and 1% methylcellulose solution.

In the CFC and CuFC, the animals were divided into seven groups, seven rats in each. Six groups received ethanol according to the scheme presented above and the control group received only water.

The experiments were carried out in strict accordance with Polish governmental regulations, concerning experiments on animals (Dz.U.05.33.289). All experimental protocols were approved by the Local Ethical Committee for Experimentation on Animals (resolution no. 77/ŁB/587/2011).

### Drugs

Retigabine (Trobalt, GlaxoGroup Ltd.^®^) was administered directly to the stomach via oral gavage at a dose of 10 mg/kg. The drug was given as suspension in 1% aqueous methylcellulose solution in the amount of 0.2 ml/100 g. Retigabine was given only to animals from the experimental group in the MWM. During the MWM trials days, the animals received the drug after the end of the test in order to avoid the influence of the acute dose of the drug on the obtained results. The effect of retigabine administered alone on memory processes was assessed in MWM in our earlier study [16]. In the CFC and CuFC, the drug was administered together with ethanol for 3 weeks and also in the fourth week (without alcohol) only to two groups (RTG/ET_3W + RTG_4W). The other two groups (ET_3W + RTG_4W) received the retigabine only in the fourth week of the experiment (after the discontinuation of ethanol administration). One group receiving the drug according to the selected schedule underwent the study after 24 h and the other after 72 h from discontinuation of alcohol administration.

### Morris water maze test

The MWM is a behavioral test, used to study spatial learning and memory. It consists of a circular pool (180 cm in diameter; 50 cm high walls) filled with water (22–24 °C) and virtually divided into four sections. The pool is placed in a room with several extra-maze cues. A transparent circular platform (8 cm in diameter) which was used during the experiment, was placed in the center of the selected quadrant about 2 cm below the surface of the water and it was invisible for animals. The experiments were recorded with a camera hung above the pool, which allowed close monitoring of the test in real-time without eye contact between the animal and the researcher. Rat position, movement, and entrance to the platform were detected using ANY-maze software (ANY-maze, USA).

The rats were pre-trained to learn the experiment scheme during the first 3 days of the experiment. The platform had been placed in a selected quadrant before the animals were introduced. Each time, the animal was put into the pool in a different quadrant, facing the wall, and allowed to swim for 60 s. If the rat managed to find the platform, it was allowed to stay on it for 15 s and observe the room. If the animal did not find the platform within the 60 s, it was placed there manually by the experimenter for 15 s to observe the room. The experiment was repeated for each animal four times per day with 60 s intervals between trials.

After 3 days of pre-training, the retention test was performed in an analogous scheme but without the hidden platform. During these trials, the animals spent more time swimming in the quadrant that previously contained the platform and an inability to locate it in the memorized place caused that the rats search for it along with the entire pool.

MWM was performed after the first and third week of ethanol administration and in the first week after the discontinuation of ethanol administration. The platform was moved to another quadrant each week, and the activities of days 1–4 were repeated. The platform was in the same place every 1–4 trials days. During the alcohol administration period, MWM trials were performed about 3 h after administration of morning low dose of ethanol to prevent the possible effect of an acute dose of alcohol on animal behavior. For the same reason, retigabine was administered after rat’s swimming on the MWM trials days.

### Contextual fear conditioning

The CFC is a basic behavioral test to study associative learning. The test assesses the ability of animals to associate the aversive stimulus with the environment. After returning to familiar surroundings, the rat, remembering the unpleasant experience, demonstrates a freezing response which is a fear response, defined as a lack of any activity except for breathing.

Fear conditioning system (Ugo Basile, Italy) was used in the study. It consists of a sound-attenuating chamber (55 × 60 × 57 cm), equipped with a light and speaker, and an animal box with electrified floor (30 × 34 ×  41.5 cm). Animal behavior was monitored and recorded using a camera located in the central part of the chamber.

The study lasts 2 days. The first day involves a training session and it starts with 120 s adaptation period during which the animal examines the new environment. Then, the rat is exposed to a sound signal (conditioned stimulus – CS) at a level of approximately 80 dB for 30 s. 0.5 mA footshock (unconditioned stimulus – US) is given to the animal during the last 2 s of the sound. After a 120 s break, another analogous trial is performed. After this time, the rat remains for about 60 s in the cage to associate and consolidate information. The chamber is cleaned with isopropyl alcohol in the periods in which the rats remain outside to retain the same fragrance.

After 24 h, the animal is placed in the cage and the procedure is conducted under similar conditions (light, scent, time of the test). The animal remains there for 240 s during which the total time of freezing responses is assessed. The rats are not exposed to CS or US this day. As on the previous day, the cage is cleaned with isopropyl alcohol between trials.

The CFC was performed after 24 h and 72 h following the discontinuation of ethanol administration.

### Cued fear conditioning

The CuFC is one of the basic studies evaluating associative learning in animals and it is similar to the CFC. The only difference is the procedure conducted on the second day of the study, when the animal is placed in the new environment and exposed to a known sound stimulus. Rats will be learning in this study not only to the context but especially to the tone. Similar to the CFC, the footshock is not used on the second day of the experiment.

This study involved the animals which had been used in the CFC. On the first day, the procedure was carried out similar to the CFC test. On the second day, the test was carried out 3 h after completing the CFC. The appearance of the floor and walls was changed and between trials, the cage was cleaned with isopropyl alcohol with the addition of vanilla extract. Initially, the rat was placed in the device for 180 s for adaptation. Then, the animals were exposed to a familiar sound stimulus (80 dB) for 3 min and the total time of freezing responses was evaluated. Afterward, the sound was turned off and the animal remained in the cage for another minute.

The study was performed after 24 h and 72 h following the discontinuation of ethanol administration in the device used in CFC.

### Analysis of results

The results are presented as median (horizontal bar), first and third quartiles (vertical column), and minimum and maximum (vertical line). Outliers and extreme values are represented with circles and asterisks. The normality of the distribution was checked by the Kolmogorov–Smirnov test, with Lilliefors correction. Non-parametric tests were chosen due to the abnormality of the distribution. In the MWM, a statistical analysis was performed with the Kruskal–Wallis (ANOVA) test (comparison between groups), with treatment as a between-subject factor or with post hoc Friedman test (comparison within a group), with treatment as a between-subject factor and days of trials as a repeated measure. In the CFC and CuFC, a statistical analysis was performed with the Kruskal–Wallis (ANOVA) test, with treatment as a between-subject factor. A *p* value of 0.05 or lower indicated a statistically significant difference for all statistical tests. The statistical analysis was performed using the Statistics 13.

## Results

The animals were exposed to a similar dose of ethanol. All animals from ethanol and experimental groups received 20% ethanol via oral gavage twice a day. Moreover, rats drank a similar amount of alcohol during the period of free access. The animals from the experimental group drank daily on average 21.38 ± 5.23 ml of ethanol and the rats from the ethanol group drank on average 22.85 ± 4.71 ml of ethanol. Free-ethanol consumption rate was 3.09 ± 0.71 g/kg/day in experimental group (retigabine and ethanol) and 3.26 ± 0.74 g/kg/day in ethanol group.

### Initial training before retigabine administration in the MWM

**Fig. 1:**
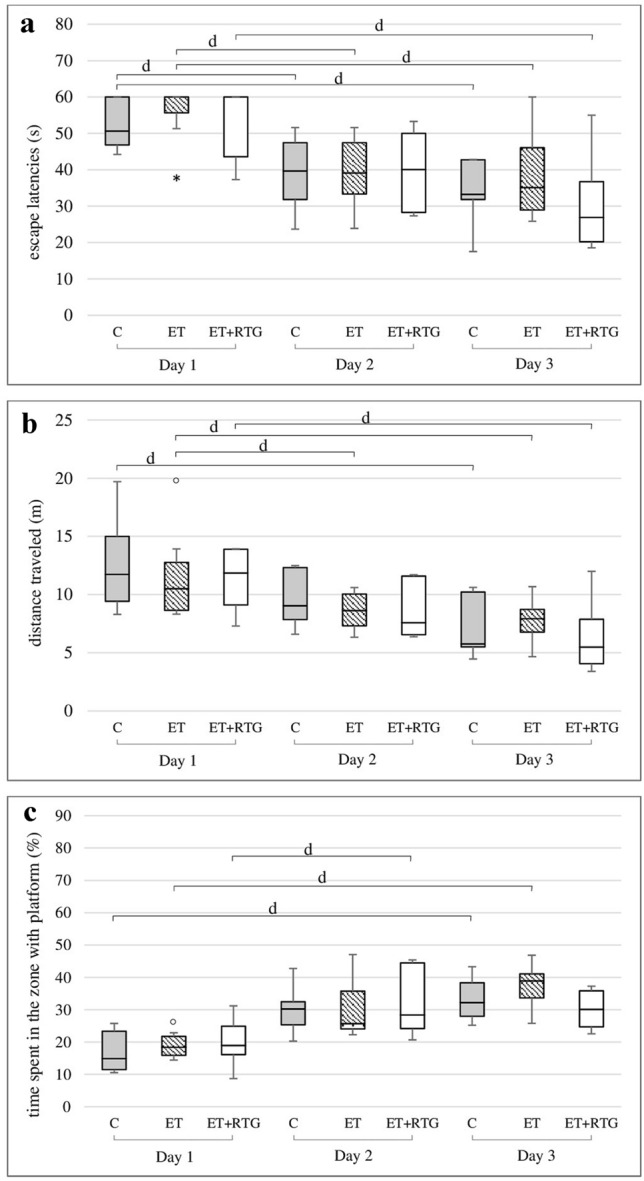
Training (before ethanol and retigabine administration): (**a**)—the time needed to localize the platform, (**b**)—the distance travelled by rats in order to localize the platform, (**c**)—the time spent in the zone with platform; C – control group, ET – ethanol group, ET + RTG – retigabine and ethanol group. ^d^Statistically significant difference between particular test day and test day 1; *p* < 0.05, *post hoc analysis for the Friedman’s test*. The results are presented as median (horizontal bar), first and third quartiles (vertical column) and minimum and maximum (vertical line). Outliers and extreme values are marked with circles and asterisks.

There was no differences between groups of the animal during training. All rats demonstrated that they need less time and shorter distance to find the platform (Fig. 1a, b). Animals spent also more time in the zone with the platform on each consecutive day of the training (Fig. 1c).

### The effect of 1-week co-administration of retigabine and ethanol on the spatial memory in rats in the MWM

**Fig. 2:**
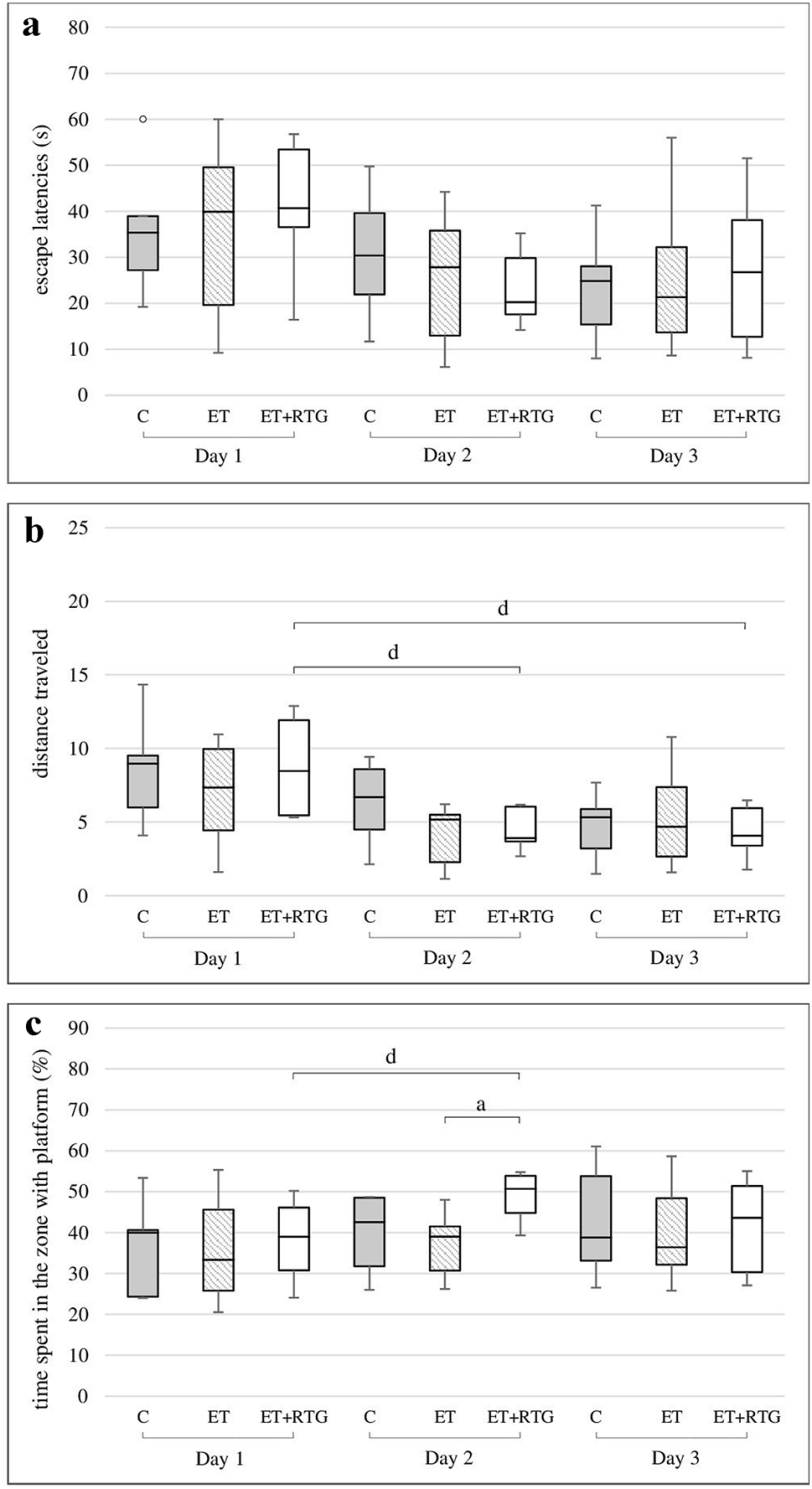
Effect of retigabine in MWM after 1 week of alcohol administration on the time needed to localize the platform (**a**), the distance travelled by rats in order to localize the platform (**b**), the time spent in the zone with platform (**c**) and after 3 weeks of alcohol administration on the time needed to localize the platform (**d**), the distance travelled by rats in order to localize the platform (**e**), the time spent in the zone with platform (**f**); C – control group, ET – ethanol group, ET + RTG – retigabine and ethanol group. ^a^Statistically significant difference between ET and ET + RTG on that day; *p* < 0.05, *Kruskal–Wallis test.* ^b^Statistically significant difference between ET and C on that day; *p* < 0.05, *Kruskal–Wallis test*. ^d^Statistically significant difference between particular test day and test day 1; *p* < 0.05, *post hoc analysis for the Friedman’s test*. The results are presented as median (horizontal bar), first and third quartiles (vertical column) and minimum and maximum (vertical line). Outliers are marked with circles.

After 1-week administration of retigabine and ethanol, there were no differences between groups in the time needed to find the platform (Fig. 2a). The distance that rats travelled was significantly shorter on consecutive days in the group of animals receiving retigabine and ethanol (Chi sq = 15.36, df = 2, *p* = 0.00046) (Fig. 2b). On the second day of the experiment, rats from the retigabine group spent significantly more time close to the platform than animals receiving only ethanol *H* = 8.512, *N*_1_ = 7, *N*_2_ = 8, *N*_3_ = 7 (*p* = 0.0142) (Fig. 2c). Moreover, retigabine significantly increased this time on the second day in comparison to the first test day (Chi sq = 6.9, df = 2, *p* = 0.0316).

### The effect of 3-week co-administration of retigabine and ethanol on the spatial memory in rats in the MWM

**Fig. 3:**
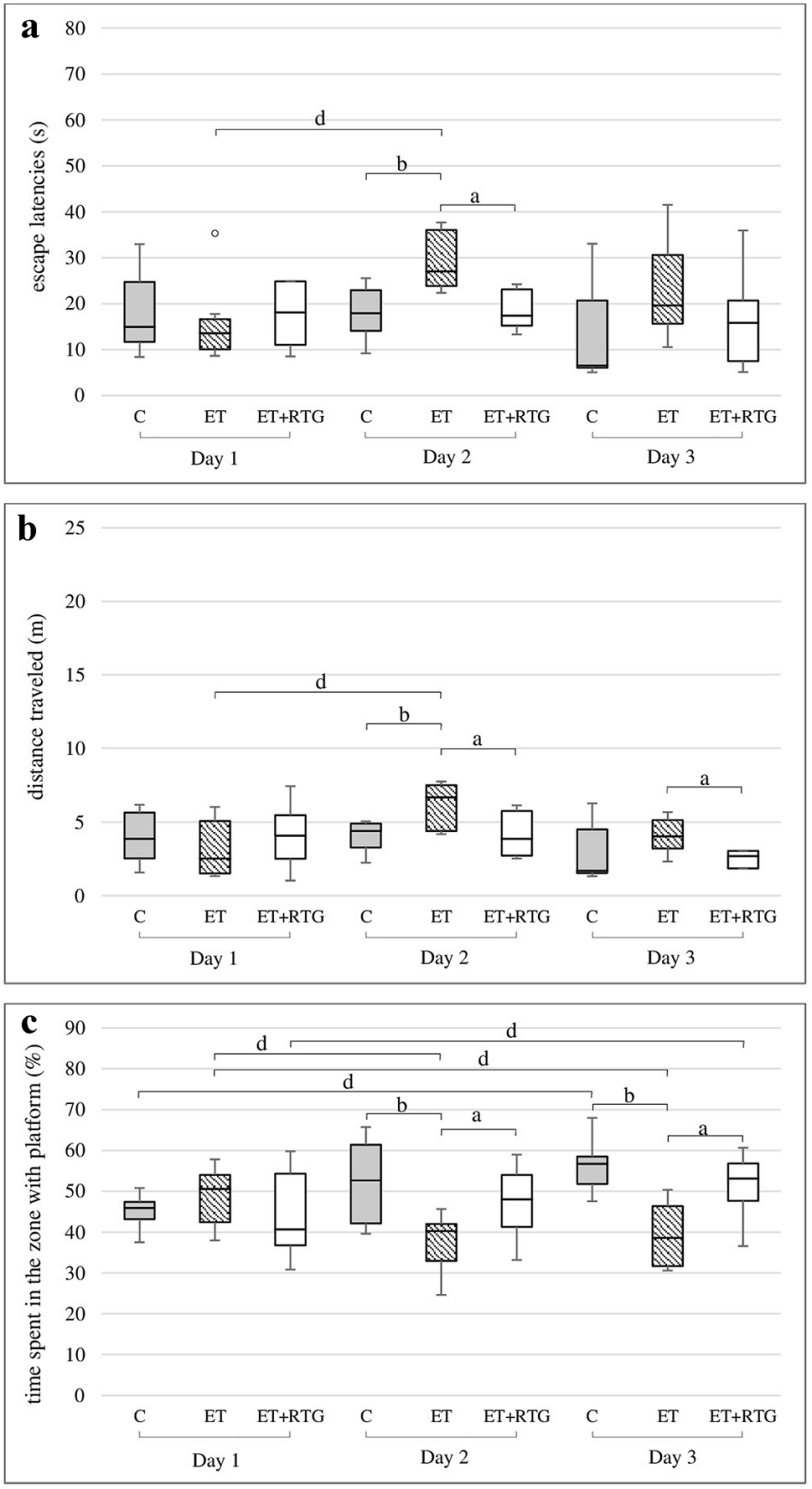
Effect of retigabine in MWM after 3 weeks of alcohol administration on the time needed to localize the platform (**a**), the distance travelled by rats in order to localize the platform (**b**), the time spent in the zone with platform (**c**); C – control group, ET – ethanol group, ET + RTG – retigabine and ethanol group. ^a^Statistically significant difference between ET and ET + RTG on that day; *p* < 0.05, *Kruskal–Wallis test*. ^b^Statistically significant difference between ET and C on that day; *p* < 0.05, *Kruskal–Wallis test*. ^d^Statistically significant difference between particular test day and test day 1; *p* < 0.05, *post hoc analysis for the Friedman’s test*. The results are presented as median (horizontal bar), first and third quartiles (vertical column) and minimum and maximum (vertical line). Outliers are marked with circles.

Ethanol administered for 3 weeks significantly prolonged the time needed to find the platform on the second and third day of the experiment (Chi sq = 6.9, df = 2, *p* = 0.0316) (Fig. 3a). Significant differences were also observed on the second test day, compared to both retigabine and control groups (*H* = 9.49, *N*_1_ = 7, *N*_2_ = 8, *N*_3_ = 7, *p* = 0.0087). Furthermore, animals which received retigabine and ethanol travelled a significantly shorter distance to find the platform in comparison to the ethanol group on the second and third test day (*H* = 6.86, *N*_1_ = 7, *N*_2_ = 8, *N*_3_ = 7, *p* = 0.0323) (Fig. 3b). A significant difference in travelled distance between the ethanol and control groups was only observed on the second day of the experiment. Animals from the ethanol group travelled a longer distance on each consecutive day to find the platform, but a significant difference was only noted between the first and second test days (Chi sq = 13.09, df = 2, *p* = 0.00144). Additionally, a significant increase in the time spent in the zone with the platform was observed on the second and third day of the study in animals receiving retigabine and ethanol as well as in the control group, compared to rats exposed only to ethanol (*H* = 8.16, *N*_1_ = 7, *N*_2_ = 8, *N*_3_ = 7, *p* = 0.0169) (*H* = 12.25, *N*_1_ = 7, *N*_2_ = 8, *N*_3_ = 7, *p* = 0.0022) (Fig. 3c). In comparison to the first test day, a gradual significant decrease in time was observed in the rats obtained ethanol on the second and third day of the experiment (Chi sq = 3.36, df = 2, *p* = 0.0186). In turn, rats from retigabine and control groups spent more time in the zone with the platform on each consecutive day but significant differences were only observed between the first and third test days (Chi sq = 3.36, df = 2, *p* = 0.0186).

### The effect of retigabine on the spatial memory in rats in the MWM after discontinuation of ethanol administration

**Fig. 4:**
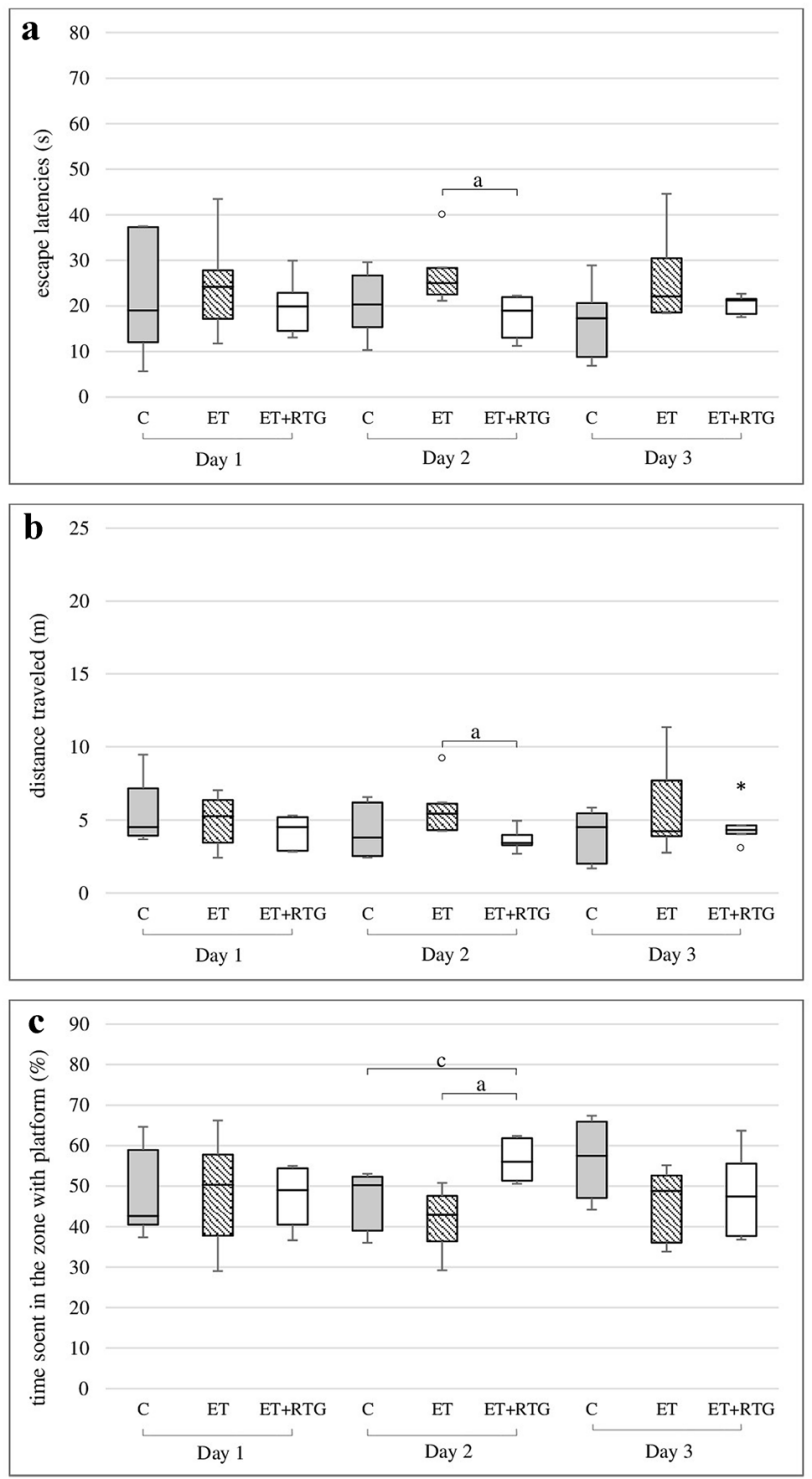
Effect of retigabine in MWM after 1 week from the discontinuation of ethanol administration on the time needed to localize the platform (**a**), the distance travelled by rats in order to localize the platform – (**b**), the time spent in the zone with platform (**c**); C – control group, ET – ethanol group, ET + RTG – retigabine and ethanol group. ^a^Statistically significant difference between ET and ET + RTG on that day; *p* < 0.05, *Kruskal–Wallis test*. ^c^Statistically significant difference between C and ET + RTG on that day; *p* < 0.05, *Kruskal–Wallis test*. The results are presented as median (horizontal bar), first and third quartiles (vertical column) and minimum and maximum (vertical line). Outliers and extreme values are marked with circles and asterisks.

Retigabine administered at a dose of 10 mg/kg decreased the time needed to find the platform on the second day of the experiment (*H* = 6.68, *N*_1_ = 7, *N*_2_ = 8, *N*_3_ = 7, *p* = 0.0354) (Fig. 4a). This effect was observed in comparison to animals receiving previously only ethanol. Moreover, on the same day, retigabine significantly reduced distance compared to rats obtained previously ethanol (*H* = 6.25, *N*_1_ = 7, *N*_2_ = 8, *N*_3_ = 7, *p* = 0.0438) (Fig. 4b). Retigabine also increased the time spent in the zone with the platform in relation to animals receiving previously ethanol and control group (*H* = 11.40, *N*_1_ = 7, *N*_2_ = 8, N_3_ = 7, *p* = 0.0033) (Fig. 4c).

### The effect of retigabine on the rats learning ability in CFC after 24 h from discontinuation of ethanol administration

**Fig. 5:**
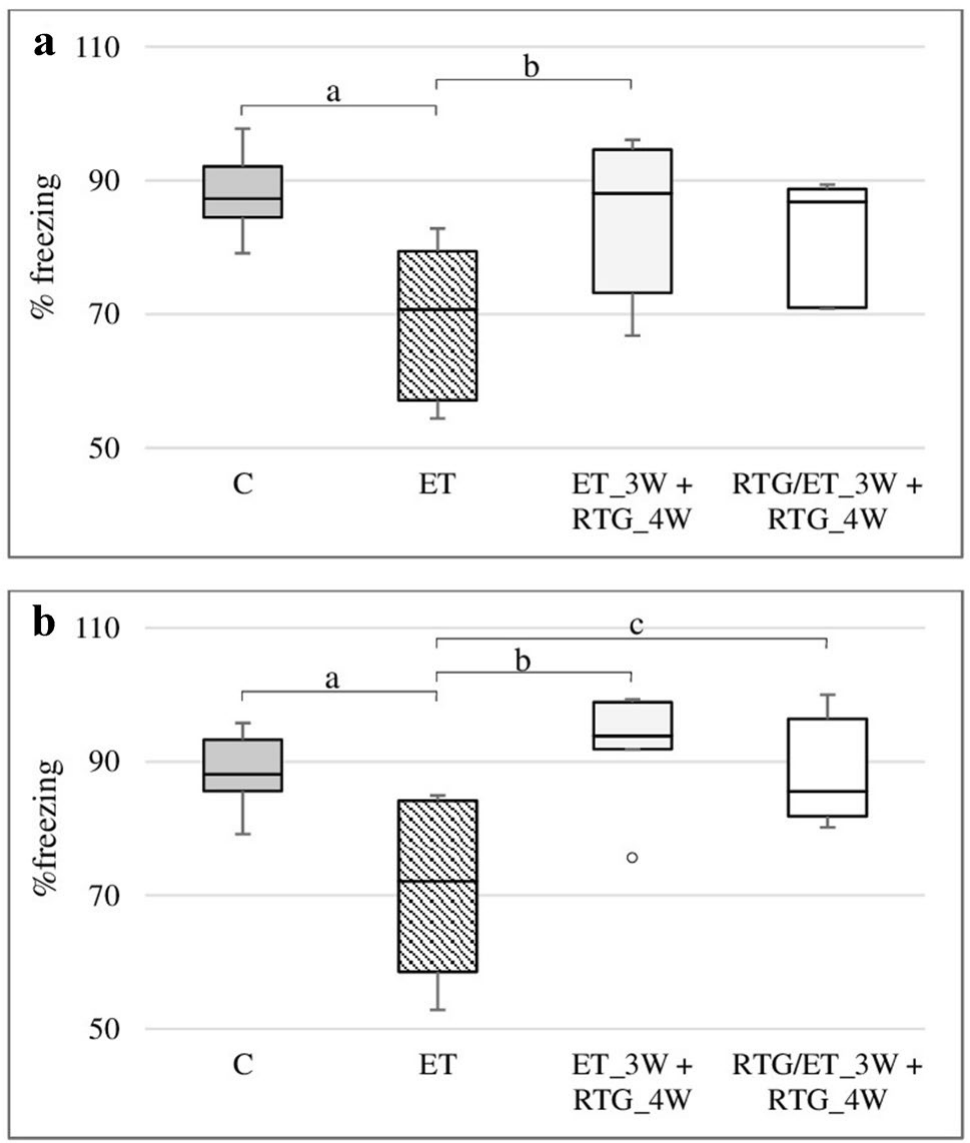
% freezing after 24 h (**a**) and 72 h (**b**) of abstinence in the CFC. ^a^Statistically significant difference between ET and C on that day; *p* < 0.05, *Kruskal–Wallis test.*^b^Statistically significant difference between ET and ET_3W + RTG_4W on that day; *p* < 0.05, *Kruskal–Wallis test.* ^c^Statistically significant difference between ET and RTG/ET_3W + RTG_4W on that day; *p* < 0.05, *Kruskal–Wallis test.* The results are presented as median (horizontal bar), first and third quartiles (vertical column) and minimum and maximum (vertical line). Outliers are marked with circles.

Retigabine administered at a dose of 10 mg/kg prolonged the time in which animals remained motionless (freezing response), comparing to rats previously receiving only ethanol (Fig. 5a). However, significant differences were observed in rats that obtained the drug only after the discontinuation of ethanol administration and also in the control group (*H* = 10.22, *N*_1_ = 7, *N*_2_ = 7, *N*_3_ = 7, *N*_4_ = 7, *p* = 0.0168).

### The effect of retigabine on the rats learning ability in CFC after 72 h from discontinuation of ethanol administration

Retigabine increased the proportion of freezing responses after 72-h from discontinuation of ethanol administration compared to animals which previously obtained only ethanol and received their last dose of ethanol also 72 h prior to testing (Fig. 5b). Significant differences were observed in both groups of rats receiving retigabine and in the control group (*H* = 10.27, *N*_1_ = 7, *N*_2_ = 7, *N*_3_ = 7, *N*_4_ = 7, *p* = 0.0164).

### The effect of retigabine on the rats learning ability in CuFC after 24 h from discontinuation of ethanol administration

**Fig. 6:**
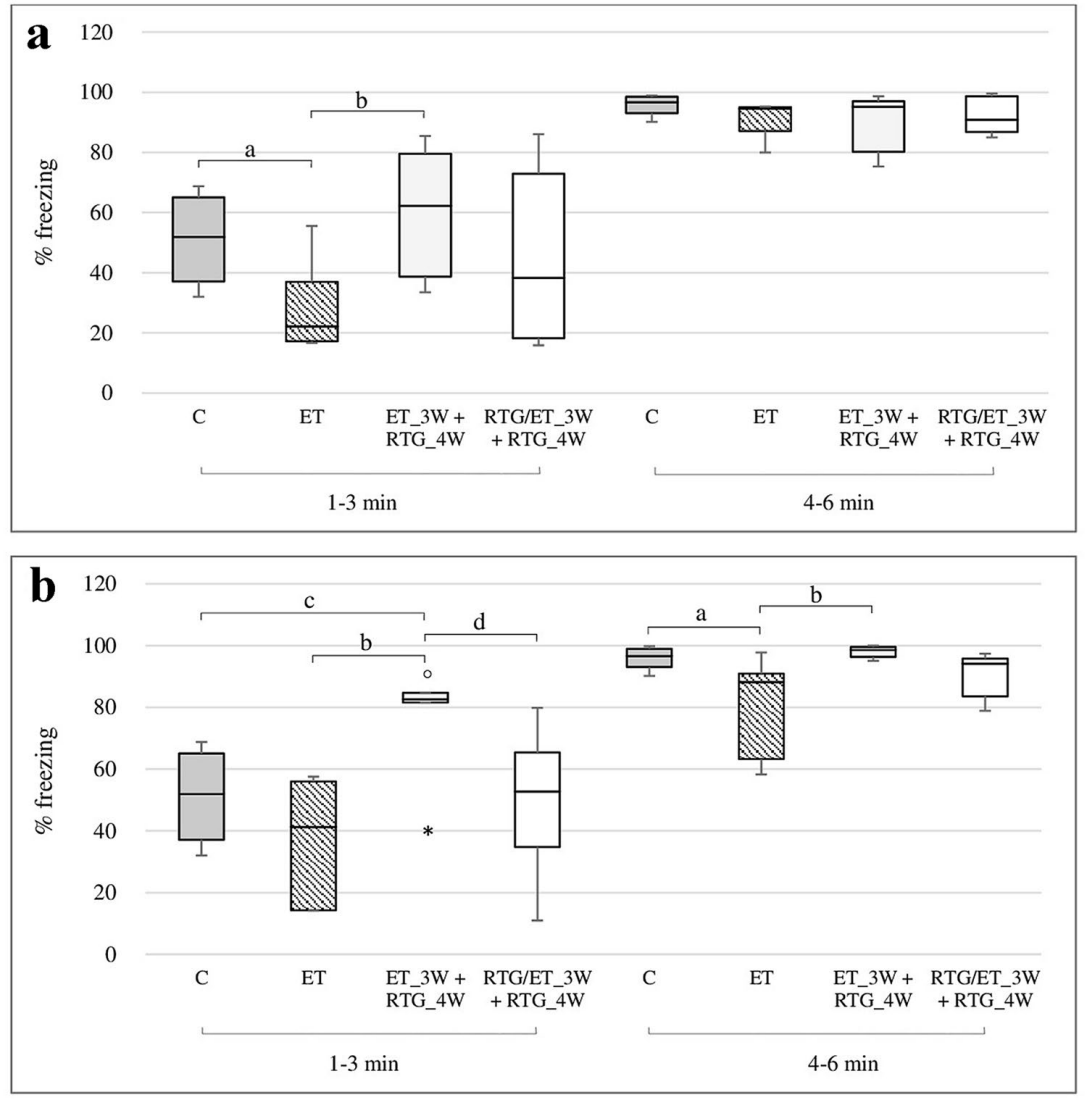
% freezing after 24 h (**a**) and 72 h (**b**) of abstinence in the CuFC. ^a^Statistically significant difference between ET and C on that day; *p* < 0.05, *Kruskal–Wallis test.* ^b^Statistically significant difference between ET and ET_3W + RTG_4W on that day; *p* < 0.05, *Kruskal–Wallis test.* ^c^Statistically significant difference between C and ET_3W + RTG_4W on that day; p < 0.05, *Kruskal–Wallis test.* ^d^Statistically significant difference between ET_3W + RTG_4W and ET/RTG_3W + RTG_4W on that day; *p* < 0.05, *Kruskal–Wallis test.* The results are presented as median (horizontal bar), first and third quartiles (vertical column) and minimum and maximum (vertical line). Outliers and extreme values are marked with circles and asterisks.

In the first 3 min of the study, the rats got acquainted with a new environment without a sound cue (Fig. 6a). Those animals which received earlier only ethanol demonstrated the highest motor activity. Significant differences were observed in comparison to animals from the control group and those receiving retigabine only after discontinuation of ethanol administration (*H* = 8.53, *N*_1_ = 7, *N*_2_ = 7, *N*_3_ = 7, *N*_4_ = 7, *p* = 0.0362).

In the next 3 minutes of the experiment during which the animals were exposed to the conditioned stimulus (sound), all animals demonstrated increased freezing behavior and there was no significant differences between groups.

### The effect of retigabine on the rats learning ability in CuFC after 72 h from discontinuation of ethanol administration

It was observed in the first stage of the study (without conditioned stimulus) that retigabine administered only after discontinuation of ethanol administration significantly increased freezing responses compared to all other studied groups (*H* = 11.47, *N*_1_ = 7, *N*_2_ = 7, *N*_3_ = 7, *N*_4_ = 7, *p* = 0.0094) (Fig. 6b).

In the next stage of the experiment, a significant increase in freezing time as a response to sound stimulus was observed. The lowest number of freezing responses was observed in rats which previously obtained only ethanol and a significant differences were noted compared to the control group and rats receiving retigabine after discontinuation of ethanol administration (*H* = 13.29, *N*_1_ = 7, N_2_ = 7, *N*_3_ = 7, *N*_4_ = 7, *p* = 0.004).

## Discussion

Retigabine is an antiepileptic drug which mechanism of action is associated with the activation of voltage-gated potassium channels Kv7 [1]. These channels regulate neural excitability and have shown to be acutely sensitive to blocking by ethanol [17]. Moreover, recent study indicates that increased excitatory activity may result in Kv7.2 upregulation in the hippocampus [18]. It is also known that ethanol may affect the hippocampus and learning-related synaptic plasticity [12]. Hence, the aim of this study was to assess the influence of retigabine on hippocampal-dependent memory processes in rats receiving ethanol in various experimental models.

The MWM was one of the used research methods. According to the theory by Redish and Touretzky [19], the hippocampus which is a brain structure associated with spatial memory plays the main role in finding hidden platforms. After the first week of alcohol administration, memory disorders were not observed in animals probably due to the short period of ethanol exposure. However, memory disturbances were observed in rats after the third week of alcohol administration, and retigabine significantly decreased this adverse effect.

It has been demonstrated in a similar behavioral model that retigabine administered to rats at a dose of 8 mg/kg *ip* ameliorates stress-induced disturbances of spatial memory. The authors suggested that this beneficial effect may be associated with regulating enzyme ubiquitin-specific proteases 2 (USP2)-related signaling pathways in hippocampal CA1 area [19]. However, in our previous study, retigabine administered as a single dose (20 mg/kg *ig*) or repeatedly (10 mg/kg *ig*), transiently disturbed learning processes in the MWM and passive avoidance test in rats [16].

The assessment of retigabine’s influence on spatial memory was continued after discontinuation of alcohol administration and it was observed that the drug slightly decreased learning impairment in this period. A significant improvement of studied parameters was observed on the second day of the experiment. It is known that people with alcohol use disorder suffer, apart from demonstrating other symptoms, also from disturbed spatial memory [20]. Hence, this effect of retigabine deserves special attention.

In the available literature, there is little information about the effect of retigabine and ethanol on the activity of the central nervous system. Knapp et al. [21] demonstrated that the drug decreases ethanol intake in rats, without affecting significantly the drinking of either water or a sucrose solution. A similar effect was observed in a two-bottle choice intermittent alcohol access paradigm in rats [11]. However, retigabine microinfused to the VTA decreased voluntary alcohol consumption in rats with a high-drinking phenotype, while the opposite effect was observed in low-drinking rats [22]. It has been noted that the gene *Kcnq2/3* that encodes Kv7 channels is associated with the regulation of ethanol consumption and prolonged drinking causes neuroadaptive changes in these channels in rats [11]. The same authors also noticed that midbrain *Kcnq4* expression negatively correlates with ethanol intake and seeking behaviors in mice and Kv7 channels in VTA regulate excessive alcohol intake in high-drinking rats [22]. The beneficial effect of retigabine on memory processes which was observed in this study may be also associated with Kv7 channels which are expressed in relation to spatial memory and navigation medial entorhinal cortex layer II stellate cells [23].

In the CFC, the effect of retigabine on memory associated with emotions and fear was assessed after 3 weeks of ethanol administration. After 24 h following the discontinuation of ethanol intake, retigabine administered only in the ethanol-free period increased freezing responses which constitutes an anxious response. The duration of immobility is closely related to the degree to which the animal associates an unpleasant stimulus with the environment and with the fear of its reappearance and this reflects hippocampal-dependent learning process. Similar effects were observed after 72 h from the discontinuation of alcohol administration. Moreover, retigabine was given both together with ethanol and only in the ethanol-free period improved emotional memory in rats, as compared to animals receiving only alcohol.

The CuFC is also a test evaluating memory associated with emotions and fear. It has been demonstrated in this study that retigabine administered only in an ethanol-free period increases freezing responses in rats in the first three minutes of the test phase when the animals were not yet exposed to sound stimulus. It may be associated with memorizing negative stimulus given on the previous day of the study, despite introducing considerable changes in the appearance of the surroundings. On the other hand, it cannot be excluded that retigabine given in an acute dose disturbs the cognitive activity of rats. In the next stage of the study, the animals were exposed to sound stimuli. All rats receiving retigabine preserved the memory of a negative stimulus but significant increases of freezing responses were observed after 72 h from discontinuation of ethanol administration between animals receiving retigabine only ethanol-free period and those who received previously only ethanol.

Tipps et al. [24] showed that withdrawal from acute exposure to ethanol may disturb learning processes but this effect was bidirectional. Contextual memory impairment was observed both in DBA/2 J mice and C57BL/6 J mice but cued learning was enhanced. In another study, acute dose of ethanol (1 or 1.5 g/kg *ip*) given before tone–shock conditioning impaired both contextual and cued learning in adult rats [25]. However, ethanol did not affect significantly emotional memory in the model of repeated withdrawn [26].

It is suggested that memory associated with contextual anxiety is dependent on the functioning of the hippocampus and prefrontal cortex [27]. The research results show that dopamine D1-like receptors in the dorsal hippocampus and basolateral amygdala contribute to the acquisition of contextual fear memory [28]. Other authors also point to the participation of the entorhinal cortex in the extinction and reconsolidation of this type of memory [29]. In in vitro and in vivo studies, retigabine inhibited the activity of mesencephalic dopaminergic systems. This effect was probably associated with the influence on Kv7.4. channels which are expressed in dopaminergic neurons in the mesolimbic and nigrostriatal pathways [30]. It has also been noted that the drug dose-dependently decreases dopamine firing rate in the ventral tegmental area. Sotty et al. [31] suggested that the activation of Kv7 potassium channels attenuates dopaminergic transmission in the mesolimbic system, particularly during its excessive activity.

No data was found in available literature about the impact of retigabine on ethanol-induced emotional memory disturbances. Only Young and Thomas [32] demonstrated that retigabine administered in a single dose may reverse the consolidation of fear memory. The drug was administered by systemic injection or infusion to the basolateral amygdala aftershock. It was observed that the drug dose-dependently inhibits freezing responses to the tone and also inhibits fear memory consolidation after bilateral infusion. The authors suggested that this activity of the drug may relieve symptoms of post-traumatic stress disorder if retigabine is administered immediately after a traumatic experience.

It is known that alcohol consumption is associated with the risk of addiction development which is now a major health problem worldwide. However, the efficacy of the drugs registered in alcohol dependence therapy is not satisfactory and the research is primarily focused on investigating substances affecting neurotransmitter systems involved in the pathogenesis of alcohol dependence. One of them is a new generation of anticonvulsants, including retigabine. Despite the previously observed effect of retigabine on reducing alcohol consumption in animals [11, 21], the drug’s mechanism of action is not fully understood. Alcohol abuse disrupts the function of the hippocampus and impairs synaptic plasticity in this structure. This impact has a complex character and involves alterations in various neurotransmitter systems, for instance, glutamatergic and GABA-ergic transmissions [12, 33]. In our previous study, retigabine administered together with alcohol reduced ethanol-induced changes in the EEG recordings in rabbits and also decreased, especially in the hippocampus, neuronal hyperactivity, which is characteristic of the withdrawal syndrome [13]. The results of this study also indicate that retigabine administered together with ethanol has a beneficial impact on memory. This modulating effect of retigabine may be a relevant element of the drug’s impact on the development of addiction. The obtained results indicate the necessity to conduct further detailed studies to fully understand the mechanism of this interaction.
